# Interaction between genes and macronutrient intake on the risk of developing type 2 diabetes: systematic review and findings from European Prospective Investigation into Cancer (EPIC)-InterAct 

**DOI:** 10.3945/ajcn.116.150094

**Published:** 2017-06-07

**Authors:** Sherly X Li, Fumiaki Imamura, Zheng Ye, Matthias B Schulze, Jusheng Zheng, Eva Ardanaz, Larraitz Arriola, Heiner Boeing, Courtney Dow, Guy Fagherazzi, Paul W Franks, Antonio Agudo, Sara Grioni, Rudolf Kaaks, Verena A Katzke, Timothy J Key, Kay Tee Khaw, Francesca R Mancini, Carmen Navarro, Peter M Nilsson, N Charlotte Onland-Moret, Kim Overvad, Domenico Palli, Salvatore Panico, J Ramón Quirós, Olov Rolandsson, Carlotta Sacerdote, María-José Sánchez, Nadia Slimani, Ivonne Sluijs, Annemieke MW Spijkerman, Anne Tjonneland, Rosario Tumino, Stephen J Sharp, Elio Riboli, Claudia Langenberg, Robert A Scott, Nita G Forouhi, Nicholas J Wareham

**Affiliations:** 1 Medical Research Council (MRC) Epidemiology Unit, University of Cambridge, Cambridge, United Kingdom;; 2 Department of Molecular Epidemiology, German Institute of Human Nutrition Potsdam-Rehbruecke, Nuthetal, Germany;; 3 German Center for Diabetes Research (DZD), Düsseldorf, Germany;; 4 Navarre Public Health Institute (ISPN), Pamplona, Spain;; 5 Center for Biomedical Research in Network Epidemiology and Public Health (CIBERESP), Madrid, Spain;; 6 Public Health Division of Gipuzkoa, San Sebastian, Spain;; 7 Bio-Donostia Institute, Basque Government, San Sebastian, Spain;; 8 French National Institute of Health and Medical Research (INSERM) U1018, Institut Gustave Roussy, Center for Research in Epidemiology and Population Health (CESP), Villejuif, France;; 9 University Paris-Saclay, University Paris-Sud, Villejuif, France;; 10 Lund University, Malmö, Sweden;; 11 Umeå University, Umeå, Sweden;; 12 Catalan Institute of Oncology (ICO), Barcelona, Spain;; 13 Epidemiology and Prevention Unit, Milan, Italy;; 14 Division of Cancer Epidemiology, German Cancer Research Center (DKFZ), Heidelberg, Germany;; 15 University of Oxford, Oxford, United Kingdom;; 16 Department of Public Health and Primary Care, University of Cambridge, Cambridge, United Kingdom;; 17 Department of Epidemiology, Murcia Regional Health Council, Biomedical Research Institute of Murcia (IMIB)-Arrixaca, Murcia, Spain;; 18 Unit of Preventive Medicine and Public Health, School of Medicine, University of Murcia, Murcia, Spain;; 19 University Medical Center Utrecht, Utrecht, Netherlands;; 20 Section for Epidemiology, Department of Public Health, Aarhus University, Aarhus, Denmark;; 21 Aalborg University Hospital, Aalborg, Denmark;; 22 Cancer Research and Prevention Institute (ISPO), Florence, Italy;; 23 Department of Clinical Medicine and Surgery, Federico II University, Naples, Italy;; 24 Public Health Directorate, Asturias, Spain;; 25 Unit of Cancer Epidemiology, City of Health and Science Hospital, University of Turin, Torino, Italy;; 26 Center for Cancer Prevention (CPO), Torino, Italy;; 27 Human Genetics Foundation (HuGeF), Torino, Italy;; 28 Andalusian School of Public Health, Granada, Spain;; 29 Biosanitary Research Institute of Granada (Granada.ibs), Granada, Spain;; 30 International Agency for Research on Cancer, Lyon, France;; 31 National Institute for Public Health and the Environment (RIVM), Bilthoven, Netherlands;; 32 Danish Cancer Society Research Center, Copenhagen, Denmark;; 33 Provincial Healthcare Company (ASP) Ragusa, Vittoria, Italy; and; 34 School of Public Health, Imperial College London, London, United Kingdom

**Keywords:** macronutrient, diet, gene, diabetes, interaction, effect modification, systematic review, replication

## Abstract

**Background:** Gene-diet interactions have been reported to contribute to the development of type 2 diabetes (T2D). However, to our knowledge, few examples have been consistently replicated to date.

**Objective:** We aimed to identify existing evidence for gene-macronutrient interactions and T2D and to examine the reported interactions in a large-scale study.

**Design:** We systematically reviewed studies reporting gene-macronutrient interactions and T2D. We searched the MEDLINE, Human Genome Epidemiology Network, and WHO International Clinical Trials Registry Platform electronic databases to identify studies published up to October 2015. Eligibility criteria included assessment of macronutrient quantity (e.g., total carbohydrate) or indicators of quality (e.g., dietary fiber) by use of self-report or objective biomarkers of intake. Interactions identified in the review were subsequently examined in the EPIC (European Prospective Investigation into Cancer)-InterAct case-cohort study (*n* = 21,148, with 9403 T2D cases; 8 European countries). Prentice-weighted Cox regression was used to estimate country-specific HRs, 95% CIs, and *P*-interaction values, which were then pooled by random-effects meta-analysis. A primary model was fitted by using the same covariates as reported in the published studies, and a second model adjusted for additional covariates and estimated the effects of isocaloric macronutrient substitution.

**Results:** Thirteen observational studies met the eligibility criteria (*n* < 1700 cases). Eight unique interactions were reported to be significant between macronutrients [carbohydrate, fat, saturated fat, dietary fiber, and glycemic load derived from self-report of dietary intake and circulating n–3 (ω-3) polyunsaturated fatty acids] and genetic variants in or near transcription factor 7–like 2 (*TCF7L2*), gastric inhibitory polypeptide receptor (*GIPR*), caveolin 2 (*CAV2*), and peptidase D (*PEPD*) (*P*-interaction < 0.05). We found no evidence of interaction when we tried to replicate previously reported interactions. In addition, no interactions were detected in models with additional covariates.

**Conclusions:** Eight gene-macronutrient interactions were identified for the risk of T2D from the literature. These interactions were not replicated in the EPIC-InterAct study, which mirrored the analyses undertaken in the original reports. Our findings highlight the importance of independent replication of reported interactions.

## INTRODUCTION

Diabetes prevention is a global public health priority (1). Type 2 diabetes (T2D) arises after insulin secretory function fails to maintain normoglycemia in the face of insulin resistance, often secondary to obesity (2). Several large randomized controlled trials demonstrated that physical activity and dietary interventions can minimize the risk of or delay the onset of T2D (3–5). Beyond these lifestyle factors, genetic variation also plays a role in the risk of T2D, and >70 genomic loci have been implicated in its etiology (6). Some investigators speculate that the identification of gene-environment interactions (particularly gene-diet interactions) might enable “personalized diets” aimed at stratifying dietary interventions by genetic factors (7), as recently implemented based on other biological variables such as the gut microbiome (8).

Among dietary factors, intake of macronutrients (carbohydrate, fat, protein) has been a major focus of public health dietary guidelines worldwide. However, there is sparse confirmatory evidence for gene-macronutrient interactions for T2D. The most widely reported example is the interaction between genetic variants in or near the transcription factor 7–like 2 gene (*TCF7L2*) and dietary fiber and related dietary factors (i.e., whole-grain intake) as markers of carbohydrate quality on T2D risk (9–13). In addition to several narrative reviews (7, 14, 15), a systematic review examined lifestyle-gene interactions for T2D and highlighted the poor quality of evidence available in 2007, owing to factors such as small sample size and the use of cross-sectional designs (16). Larger prospective studies have since been published, and far more genetic loci associated with T2D risk have been identified. Furthermore, there are several important gaps in knowledge about gene-macronutrient interactions. First, past studies did not adequately control for confounding, such as by population stratification and total energy intake (17–19), or consider effects of isocaloric macronutrient substitution. Second, to our knowledge, objective biomarkers of macronutrient intake (e.g., circulating levels of PUFAs) have not yet been investigated systematically. Finally, replication is limited to date and there is potential publication bias (14).

We aimed to systematically review the literature relating to gene-macronutrient interactions and T2D, including both self-reported and objective markers of macronutrient intake and dietary fiber. In synthesizing summary evidence on interactions, our group previously demonstrated that high heterogeneity between studies prevents meaningful meta-analyses, so a narrative approach was undertaken for this review (20). We also aimed to investigate the interactions identified from a literature-based systematic review in a large prospective study, EPIC (European Prospective Investigation into Cancer)-InterAct (21), to address research gaps relating to replication, confounding, and isocaloric macronutrient substitution.

## METHODS

### Systematic review

This systematic review conformed to Meta-analysis of Observational Studies in Epidemiology guidelines proposed by Stroup et al. (22) and to Human Genome Epidemiology Network (HuGENet) guidelines (23, 24).

Studies were eligible if they reported incident or prevalent T2D as an outcome and a statistical interaction between any genetic exposure [single nucleotide polymorphisms (SNPs), genetic risk score] and macronutrient intake. Macronutrient intake included both quantity (total carbohydrate, fat, and protein intake) and indicators of quality [dietary fiber, glycemic index, glycemic load (GL), free sugars, SFAs, MUFAs, *trans* fatty acids (FAs), PUFAs, dietary cholesterol, ratio of SFAs to PUFAs, linoleic acid, α-linolenic acid, and animal and plant protein]. Whole-grain intake was not included (see **Supplemental Table 1** for details on eligibility). In this study, “macronutrient” refers to both indicators of intake (quantity and quality) and methods used to assess intake (self-report and biomarkers such as circulating n–3 PUFAs or urinary nitrogen), unless otherwise specified. Ethanol intake was not considered a macronutrient of interest in this review because we focused on essential macronutrients for physiologic function, as included in population dietary recommendations. Studies that assessed other forms of diabetes (e.g., type 1, gestational), examined nutrigenomics or quantitative glycemic traits, or investigated the interaction between gene-lifestyle interventions without macronutrient assessment were excluded.

Following a predefined protocol, electronic searches were performed using MEDLINE, EMBASE, HuGENet, and the Cochrane Library to identify studies published on or before 31 October 2015 (an example is available in **Supplemental Table 2**). To minimize publication bias, we searched the WHO International Clinical Trials Registry Platform, gray literature (e.g., GreyNet), and names of key authors and diabetes trials and we also hand-searched relevant reviews. Medical Subject Headings and specific terms (i.e., title, abstract, and key words) were also used wherever possible to ensure sensitivity within respective databases. No restrictions were placed on language, age, publication date, or study design. Authors of 3 published studies (13, 25, 26) and 1 unpublished study (clinicaltrials.gov NCT01168297) were contacted to either assess eligibility or collect further data to conduct the review. Two studies, 1 published (26) and 1 unpublished, were subsequently determined to be ineligible. Studies were screened by title and abstract for eligibility for full-text review. We extracted information from each publication meeting the eligibility criteria through the use of an agreed-upon data extraction form on cohort characteristics (e.g., study design, sample size, ethnicity, etc), covariates, statistical analyses, and estimates of associations between *1*) macronutrient intake and T2D, *2*) genetic variant and T2D, and *3*) gene-macronutrient interactions and T2D. Narrative synthesis was undertaken.

An assessment for confounding, bias (selection, measurement, attrition, outcome, and reporting), and genetic-specific issues (genotyping quality, population stratification, multiple testing) was undertaken through the use of a modified version of the Cochrane guidelines for nonrandomized studies of interventions to incorporate genetic issues highlighted by HuGENet (23, 27). This broadly classified studies as being of low, moderate, serious, or critical risk of bias.

Two authors (SXL and ZY) independently undertook every stage of screening, selection, data extraction, and quality assessment in duplicate and resolved any disagreements by discussion with 2 other authors (NGF and RAS).

### EPIC-InterAct study

To investigate the reproducibility of the statistically significant interactions identified by our systematic review, we examined them in a large-scale study (EPIC-InterAct). EPIC-InterAct participants provided informed consent and an ethics committee approved the study (21). EPIC-InterAct is a case-cohort study nested within the EPIC study (28) composed of 12,403 individuals with T2D and a randomly assigned subcohort of 16,154 individuals, as previously described (21). Data on lifestyle variables were collected from questionnaires that participants completed at baseline (from 1991). Follow-up was censored at the date of T2D diagnosis, 31 December 2007, or the date of death, whichever occurred earlier. Our current analyses were based on a smaller subset of EPIC-InterAct with available macronutrient and genome-wide genotyping data, representing 8 European countries (*n* = 9403 cases and 11,745 noncases for analyses on macronutrient intake; *n* = 9937 cases and 12,336 noncases for analyses on circulating FAs).

### DNA extraction, genotyping, and SNP selection

Methods for DNA extraction from blood samples and genotyping were previously described (21). Briefly, participant samples were genotyped on Illumina 660W-Quad BeadChip or Illumina HumanCore Exome Chip arrays (12v1 and 24v1) and were imputed to the Haplotype Reference Consortium using IMPUTE software (version 2.3.2; http://mathgen.stats.ox.ac.uk/impute/impute_v2.html). SNPs that were identified in the systematic review to significantly interact with macronutrients were carried forward for analysis in EPIC-InterAct. All SNPs met quality control criteria for the genotyping call rate (≥95%) or were well imputed (imputation accuracy information metric ≥0.99). Genotypes were in Hardy-Weinberg equilibrium.

### Self-reported and objective biomarkers of macronutrient intake

Habitual self-reported macronutrient intake data were derived from the validated self- or interviewer-administered country-specific food-frequency questionnaire (FFQ) or dietary histories taken at baseline (29, 30), with nutrient composition derived from the EPIC Nutrient DataBase (31). Baseline circulating plasma phospholipid FAs were profiled using a high-throughput automated gas chromatography method (32).

### Statistical analyses

Macronutrients were evaluated either as the percentage of total energy intake, dietary fiber as density of energy intake (1 g/1000 kcal), grams of carbohydrate for GL (previously described) (33), or the percentage of total circulating plasma phospholipid FAs. Total energy includes energy from carbohydrate, fat, protein, and alcohol intake. We assumed additive models for all genetic variants unless previously published studies demonstrated a more appropriate alternative. Multiplicative interactions between SNPs and macronutrient intake on incident T2D were analyzed using Prentice-weighted Cox regression (34) by including a product term between the SNP and macronutrient intake. Crude and multivariable-adjusted models were analyzed within countries and HRs were combined using random-effects meta-analysis to account for variation between countries. Between-country heterogeneity was assessed with Cochran’s *Q* test and *I*^2^. Each macronutrient was categorized based on the distribution of the macronutrient intake within the subcohort sample, excluding outliers (±3 SD from the mean). To account for between-country variations in dietary intake, categorization was performed per country and country-specific Cox regression was then conducted. Because categorization was performed in each country, the pooled category-specific ranges may appear to overlap. However, individuals were mutually exclusive within each category by country. Two approaches to modeling were taken: a replication model adopted the same covariates as those reported in the published study identified in the systematic review, and a modified model accounted for isocaloric macronutrient substitution and additional confounders that may bias interaction results (19) (the **Supplemental Methods** provide further rationale and description). For 2 replication analyses, we excluded EPIC-InterAct centers (Potsdam and Malmö) that contributed to previous analyses (10, 35). For example, the interaction between caveolin 2 (*CAV2*) and total fat and SFAs identified by Fisher et al. was examined in the EPIC-Potsdam study, so Potsdam was excluded from our EPIC-InterAct analysis.

We undertook complete case analyses so that those with missing macronutrient intake, genetic data, or covariates were excluded. Stata software (version 14; StataCorp LP) was used for all analyses, with a *P*-interaction < 0.05 judged as statistically significant on the basis that each interaction was considered an independent replication attempt.

## RESULTS

### Systematic review

**FIGURE 1:**
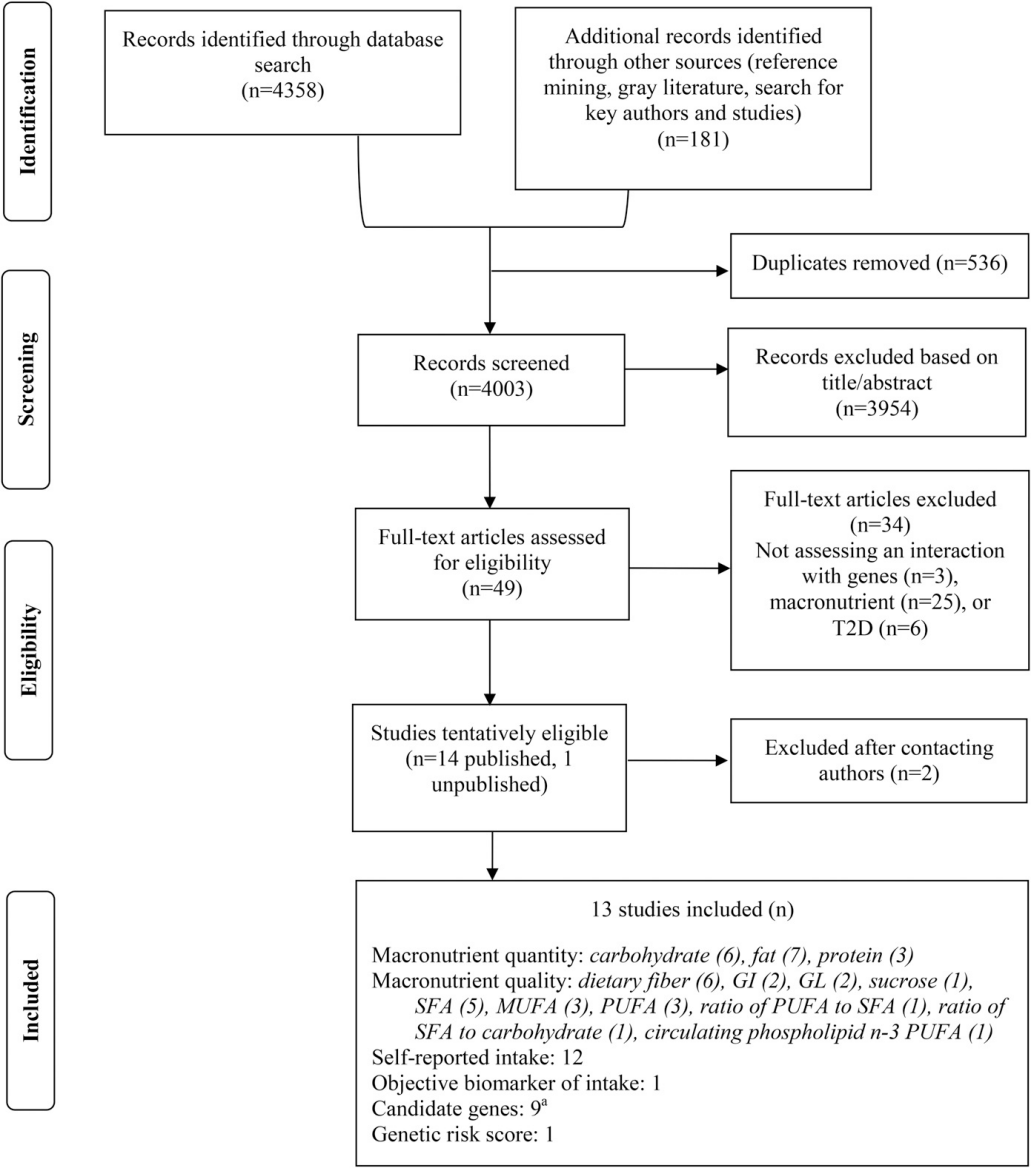
Flow diagram of the systematic review for gene-macronutrient interactions and the risk of T2D. Numbers are not mutually exclusive. ^a^This does not include exploratory studies that examined many candidate genes. GI, glycemic index; GL, glycemic load; T2D, type 2 diabetes.

Of 4003 records screened, 13 publications were included in this review (**Figure 1**). Four were cross-sectional studies (25, 36–38), 2 were case-control studies (13, 39), 1 was a family-based association study (40) and 6 were prospective (cohort or case-cohort) studies (10, 11, 35, 41–43). Study populations ranged from 805 (38) to 24,840 (41) participants (*n* = 165–1649 cases). Participants had a mean age of 50 y and were overweight on average [mean BMI (in kg/m^2^): 27]. All studies examined a self-reported diet (*n* = 12) except one, which measured erythrocyte phospholipid n–3 PUFAs (39). Across the studies examined, all macronutrients were represented except for protein quality (animal or plant protein). We examined interactions between macronutrients and SNPs from 9 candidate genetic loci [*TCF7L2*, gastric inhibitory polypeptide receptor (*GIPR*), insulin receptor substrate 1 (*IRS1*), peroxisome proliferator–activated receptor γ (*PPAR*γ), apolipoprotein A2 (*APOA2*),* CAV2*, fatty acid binding protein 1/2/3/4 (*FABP1/2/3/4*), PPARγ coactivator-1α (*PGC-1*α), and peptidase D (*PEPD*)] and a genetic risk score comprising variants in 15 T2D-associated loci (36). High heterogeneity in macronutrient categorization, genetic model, statistical interaction method, and reporting was evident.

### Gene-macronutrient interactions from the systematic review

The following 8 interactions between SNPs and macronutrients were reported to be significant: 2 SNPs in the *TCF7L2* gene with dietary fiber (10, 11), another *TCF7L2* variant with GL (13), 1 SNP in *GIPR* with total fat and carbohydrate intake (41), 1 SNP in *CAV2* with total fat and SFAs (35), and 1 SNP in *PEPD* with erythrocyte phospholipid n–3 PUFAs (39). These interactions are summarized in **Table 1** (magnitude of effects in **Supplemental Table 3**) and are described next.

**TABLE 1:** Summary of all eligible studies within the systematic review of gene-macronutrient interactions and T2D^1^

					Participants, *n*		Population characteristics						
Genetic locus	Variant	Reference	Study	Study type	Cases	Total	Country, ethnicity	Age, y	Sex, % male	BMI, kg/m^2^	Macronutrient	Interaction results	*P*-interaction
*TCF7L2*	rs7903146	Hindy et al., 2012 (10)	MDCS	Cohort	1649	24,799	Sweden, European	58.1 ± 7.6	39	25.7 ± 3.9	Total fiber, carbohydrate, fat, protein	Dietary fiber × *TCF7L2*	0.049
												↑ fiber ↓ T2D risk in CC genotype	
												↑ fiber ↑ T2D risk in T allele carriers	
	rs7903146, rs4506565	Wirström et al., 2013 (11)	SDPP	Cohort	165	5477	Sweden, European	47.2^2^	42	NA	Cereal fiber	rs7903146: ↑ fiber ↓ T2D risk in CC genotype	rs7903146: 0.005
												rs4506565: ↑ fiber ↓ T2D risk in AA genotype	rs4506565: 0.006
	rs12255372	Cornelis et al., 2009 (13)	NHS	Case-control	1140	3055	United States, European	47.5 ± 6.9	0	24.5 ± 4.6	GL, carbohydtrate, GI, cereal fiber	GL × *TCF7L2*	0.003
												Interaction disappeared after adjusting for family history	0.13
*GIPR*	rs10423928	Sonestedt et al., 2012 (41)	MDCS	Cohort	1541	24,840	Sweden, European	58 ± 7.7	39	25.7 ± 4	Carbohydrate, fat, protein, fiber, sucrose	Carbohydrate × *GIPR*	0.001
												Fat × *GIPR*	0.002
												↑ fat and ↓ carbohydrate reduces T2D in A allele carriers; composition for T allele carriers is the opposite	
*IRS1 *	rs2943641	Ericson et al., 2013 (42)	MDCS	Cohort	1567	24,841	Sweden, European	58 ± 7.7	39	25.6 ± 4	Carbohydrate	No significant interaction	0.59
											Fat		0.40
											Protein		0.28
											Fiber		0.92
	rs7578326, rs2943641	Zheng et al. (2013) (37)	GOLDN, BPRHS	Cross-sectional	419	1664	United States, Hispanic, African, European, Native American	53.5 ± 13.2	38	30.3 ± 6.4	Carbohydrate, fat, SFAs, MUFAs, SFA:carbohydrate ratio, GI, GL	No significant interaction	NA
*PPARγ*	Pro12Ala/rs1801282	Lamri et al., 2012 (43)	DESIR	Cohort	191	4676	France, European	46.8 ± 10	49	24.7 ± 3.8	Fat	No significant interaction	0.05
	1431C>T												
	Pro12Ala	Cornelis et al., 2009 (13)	NHS	Case-control	1140	3055	United States, European	47.5 ± 6.9	0	24.5 ± 4.6	Carbohydrate, GI, GL, cereal fiber	No significant interaction	NA
	Pro12Ala	Nelson et al., 2007 (40)	GENI	Family-based association analysis	736	1318	United States, Hispanic, European	40.9 ± 19.4	43	30.5 ± 6.6	PUFAs, SFAs, MUFAs, PUFA:SFA ratio	No significant interaction	NA
	Pro12Ala	Fisher et al. (2011) (35)	EPIC-Potsdam	Case-control	192	576	Germany, European	50.4 ± 8.9	42	26.7 ± 4.6	Fat	No significant interaction	0.32
	63 SNPs examined with only *CAV2* and *PPAR*γ taken forward for confirmation			Case-cohort	614	2862					SFAs		0.08
											MUFAs		0.29
											PUFAs		0.07
*APOA2*	−265T>C	Corella et al., 2011 (25)	PREDIMED and SNHS	Cross-sectional	825	2830	Singapore, Asian; Spain, European	44.1 ± 16	41	25 ± 5.3	SFAs	No interaction reported	NA
*CAV2*	rs2270188; 63 SNPs examined	Fisher et al. (2011) (35)	EPIC-Potsdam	Case-control	192	576	Germany, European	50.4 ± 8.9	42	26.7 ± 4.6	Fat	Fat × *CAV2*	0.02
				Case-cohort	614	2862							
											SFAs, MUFAs, PUFAs	SFA × *CAV2*	0.002
												↑ fat and SFA ↑ T2D among TT genotype (confirmatory analyses)	
*FABP1/2/3/4*	rs2197076; 12 SNPs examined	Mansego et al., 2012 (38)	Hortega, Segovia	Replication cross-sectional	174	2022	Spain, European	52.8 ± 11.2	45	27.5 ± 4.1	Fat, SFAs, PUFAs	No significant interaction after multiple testing correction	0.03
*PGC-1α*	Gly482Ser, Thr612Met, Thr528Thr	Nelson et al. (2007) (40**)**	GENI	Family-based association analysis	736	1318	United States, Hispanic, European	40.9 ± 19.4	43	30.5 ± 6.6	PUFAs, SFAs, MUFAs, PUFA:SFA ratio	No significant interaction	NA
*PEPD*	rs3786897; 9 SNPs examined	Zheng et al. (2015) (39)	—	Case-control	622	915	China, Asian	51.1 ± 13.2	51	24.5 ± 2.7	Circulating erythrocyte membrane phospholipid n–3 PUFAs	n–3 PUFA × *PEPD*	0.027
												↓ n–3 PUFA ↑ T2D among A allele carriers	
												↑ n–3 PUFA is not associated with T2D among A allele carriers	
GRS	Based on 15 T2D genetic loci, weighted score	Villegas et al. (2014) (36)		NHANES cross-sectional	1337	13,120	United States, European	51.1 ± 13.2	51	24.5 ± 2.7	Carbohydrate	No significant interaction (non-Hispanic whites)	0.53
**											Fiber		0.09

1 Values are presented as means ± SDs unless otherwise indicated. For magnitude of effects (e.g., ORs and 95% CIs), refer to Supplemental Table 3. *APOA2*, apolipoprotein A2; BPRHS, Boston Puerto Rican Health Study; *CAV2*, caveolin 2; DESIR, Data from an Epidemiological Study on the Insulin Resistance Syndrome; EPIC, European Prospective Investigation into Cancer and Nutrition; *FAPB*, fatty acid binding protein; GENI, Gene Environment Interactions; GI, glycemic index; *GIPR*, gastric inhibitory polypeptide receptor; GL, glycemic load; GOLDN, Genetics of Lipid Lowering Drugs and Diet Network; GRS, genetic risk score; *IRS1*, insulin receptor substrate 1; MDCS, Malmö Diet and Cancer Study; NA, not available; NHS, Nurse’s Health Study; *PEPD*, peptidase D; *PGC-1*α, peroxisome proliferator–activated receptor γ coactivator-1α *PPAR*γ, peroxisome proliferator–activated receptor γ PREDIMED, Prevención con Dieta Mediterránea; SDPP, Stockholm Diabetes Prevention Program; SNHS, Singapore National Health Survey; SNP, single nucleotide polymorphism; *TCF7L2*, transcription factor 7–like 2; T2D, type 2 diabetes; ↑, increased; ↓, decreased.

2 Mean.

Several studies examined variants in or near *TCF7L2*, the common variant with the strongest association with T2D (6). In particular, the interaction with dietary fiber was the most widely examined (*n* = 4 studies), although it was inconsistently replicated. One study reported that the effect of the T allele of rs7903146 (within *TCF7L2*) on T2D risk was significantly increased with higher intakes of total dietary fiber (10), which was corroborated by another study investigating cereal fiber (11). However, the results of 2 other studies were discordant (13, 36). In addition, Cornelis et al. (13) observed another interaction among US women, in which T allele carriers demonstrated increased odds of T2D with diets higher in GL.

One study reported that carriers of the A allele for an SNP (rs10423928) within *GIPR*, a candidate gene chosen based on the hypothesis that it encodes the receptor for the incretin hormone gastric inhibitory polypeptide (41), had a lower 12-y incidence of T2D only if they also consumed a diet higher in fat or lower in carbohydrate (41).

Another study followed up *CAV2* (rs2270188) for interaction with fat intake after exploratory analysis. This gene encodes a protein found on the surface of caveolae (small invaginations of cellular plasma membranes) and may be involved in lipid metabolism. *CAV2* has not previously been associated with T2D (OR for rs2270188: 0.99; 95% CI: 0.97, 1.01; *P* = 0.49; **Supplemental Table 4**) (44). The authors found that compared with individuals with the GG genotype, those with the TT genotype had a higher risk of T2D when they consumed diets higher in total fat and SFAs (35).

In a Chinese case-control study, Zheng et al. (39) reported an interaction between circulating n–3 PUFAs and *PEPD*, which encodes a peptidase involved in proline recycling and collagen production. Within the gene *PEPD*, rs3786897 has been associated with T2D in Asians (45). Compared with those with a GG genotype, individuals with a GA or AA genotype were found to be at higher risk of T2D only among adults possessing lower levels of n–3 PUFAs (≤5.33% of total circulating phospholipid PUFAs).

Four studies investigated the interaction between FA intake and SNPs in or near *PPAR*γ on T2D risk but identified no statistically significant interactions. This was also the case for studies examining interactions with *IRS1*, *APOA2, FABP1/2/3/4*, *PGC-1*α and a T2D-associated genetic risk score.

### Assessment of risk of bias and quality of evidence

All studies included in the review were observational and rated either at moderate (*n* = 8) or serious risk of bias (*n* = 5) (see Supplemental Table 3 for more information). Of the 6 studies reporting interactions, 3 did not account for multiple testing correction (α < 0.05) when examining several macronutrients and/or SNPs (e.g., an exploratory study examining 64 SNPs with 4 FAs; a total of 256 tests) (10, 35, 41). Two studies that published a statistically significant interaction included accompanying replication results (35, 37). Many studies did not adjust for known confounders. Confounders such as total energy intake, physical activity, and population stratification were frequently ignored (39, 43, 46). Population stratification, in particular, was considered in only one study (25). Other concerns included the validity and reliability of the dietary measurement tool (11, 36, 38, 43) and possible selective analysis and reporting (25, 38).

### Findings in EPIC-InterAct

The EPIC-InterAct population used for this analysis was broadly similar to the average population characteristics of the cohorts from the systematic review. The mean age at baseline was 52.3 y and 55.7 y for noncases and cases, respectively. Participants were overweight, with a mean BMI of 25.8 and 29.7 for noncases and cases, respectively (**Supplemental Table 5**). Associations between SNPs and T2D were comparable with the previously published genome-wide meta-analysis of genetic variants for T2D (Supplemental Table 4) (44).

**FIGURE 2:**
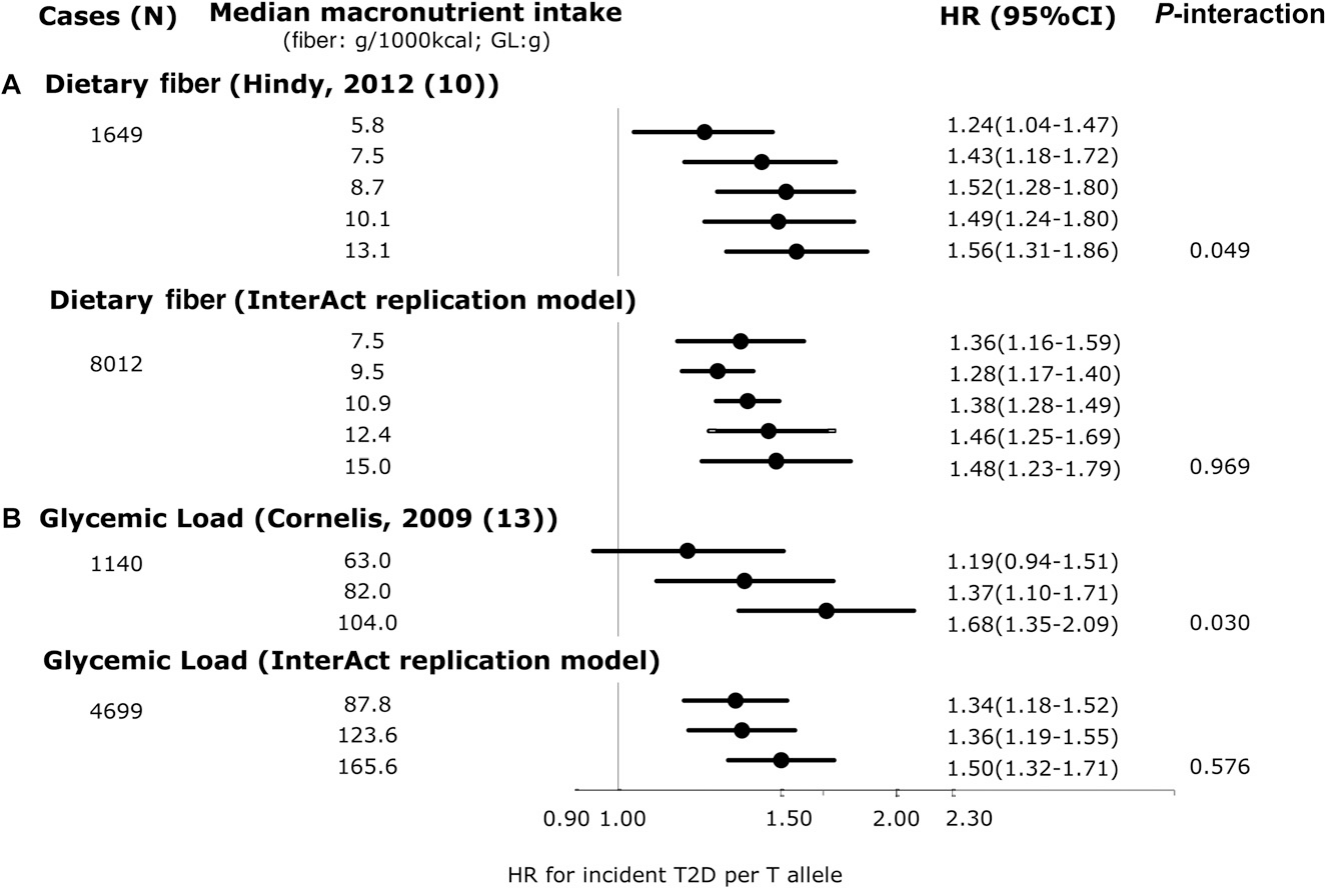
Interaction between genetic variants within *TCF7L2* and dietary fiber or GL: comparison between studies by Hindy et al. (10) and Cornelis et al. (13) with EPIC-InterAct. (A) ORs from Hindy et al. (10) (top) and pooled HRs from EPIC-InterAct (bottom) for T2D per T allele of rs7903146 (*TCF7L2*) and quintiles of dietary fiber (expressed in g/1000 kcal). Hindy et al. (10) adjusted for age, sex, BMI, total energy intake, season, and method (dietary intake assessment method). The EPIC-InterAct replication model adjusted for age (equal to the underlying time scale), sex, study center, BMI, total energy intake, and season, excluding the Malmo EPIC-InterAct center. (B) ORs from Cornelis et al. (13) and HRs from EPIC-InterAct for T2D per T allele of rs12255372 (*TCF7L2*) by tertiles of GL (in grams). Cornelis et al. (13) adjusted for age, BMI, smoking status, alcohol intake, coffee consumption, menopausal status, physical activity, energy-adjusted ratio of PUFAs to SFAs, and *trans* fat and cereal fiber intake for women only. EPIC-InterAct adjusted for age (equal to the underlying time scale), study center, BMI, smoking status, alcohol intake, coffee consumption, menopausal status, physical activity, energy-adjusted ratio of PUFAs to SFAs, and cereal fiber intake. Given that Cornelis et al. (13) evaluated this interaction in a female cohort (Nurses’ Health Study), the EPIC-InterAct analysis was conducted for women only. *P*-interaction values for EPIC-InterAct were estimated by treating macronutrients and SNPs as continuous variables. Heterogeneity between countries was not significant in the EPIC-InterAct study (*I*^2^ = 0% and 1% in panels A and B, respectively). Two SNPs (rs7903146 and rs12255372) were in moderate linkage disequilibrium (CEU, *r*^2^ = 0.7). The sample size for the EPIC-InterAct analysis of the interaction between dietary fiber and *TCF7L2* interaction was 18,292, whereas the sample size was 11,992 (women only) for the interaction between GL and *TCF7L2*. Multiplicative interaction analysis was performed with Prentice-weighted Cox regression. CEU, Northern Europeans from Utah; EPIC, European Prospective Investigation into Cancer; GL, glycemic load; SNP, single nucleotide polymorphism; T2D, type 2 diabetes; *TCF7L2*, transcription factor 7–like 2.

**FIGURE 3:**
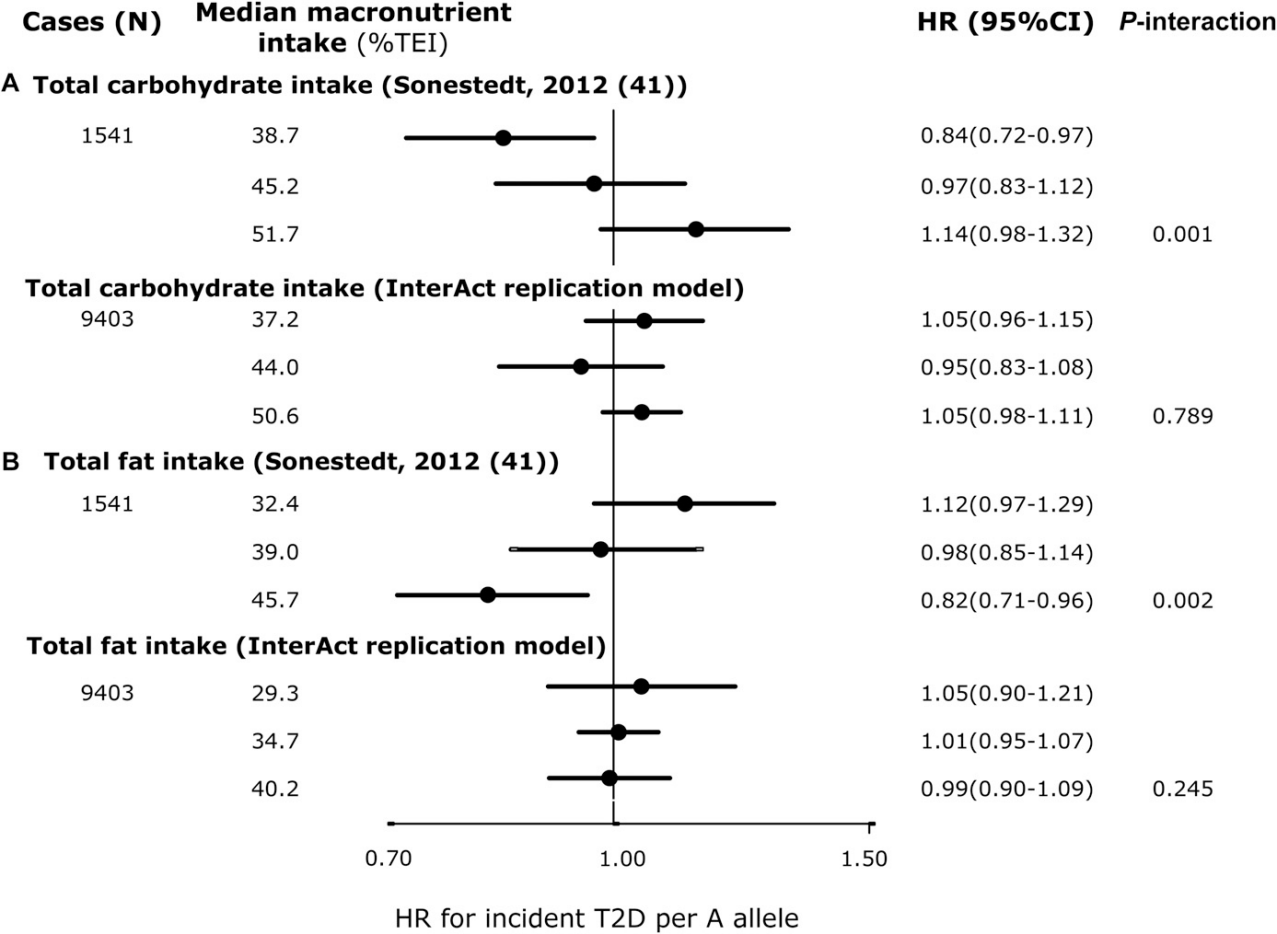
HRs of incident T2D per A allele of rs10423928 (*GIPR*) by tertiles of macronutrient intake: comparison between Sonestedt et al. (41) and EPIC-InterAct. (A and B) HRs from Sonestedt et al. (41) (top) and pooled HRs from EPIC-InterAct (bottom) for both total carbohydrate intake (A) and total fat intake (B). Sonestedt et al. (41) adjusted for age, sex, physical activity, education, smoking status, sex-specific alcohol categories, season, TEI, method, and BMI. EPIC-InterAct replication adjusted for age (equal to the underlying time scale), sex, center, physical activity, education, smoking status, sex-specific alcohol categories, season, TEI, and BMI. *P*-interaction values for EPIC-InterAct were estimated by treating macronutrients and rs10423928 as continuous variables. Heterogeneity between countries was not significant in the EPIC-InterAct study (*I*^2^ = 17% and 19% in panels A and B, respectively). The total sample size for the EPIC-InterAct analysis was 21,148. Multiplicative interaction analysis was performed with Prentice-weighted Cox regression. EPIC, European Prospective Investigation into Cancer; *GIPR*, gastric inhibitory polypeptide receptor; TEI, total energy intake; T2D, type 2 diabetes.

**FIGURE 4:**
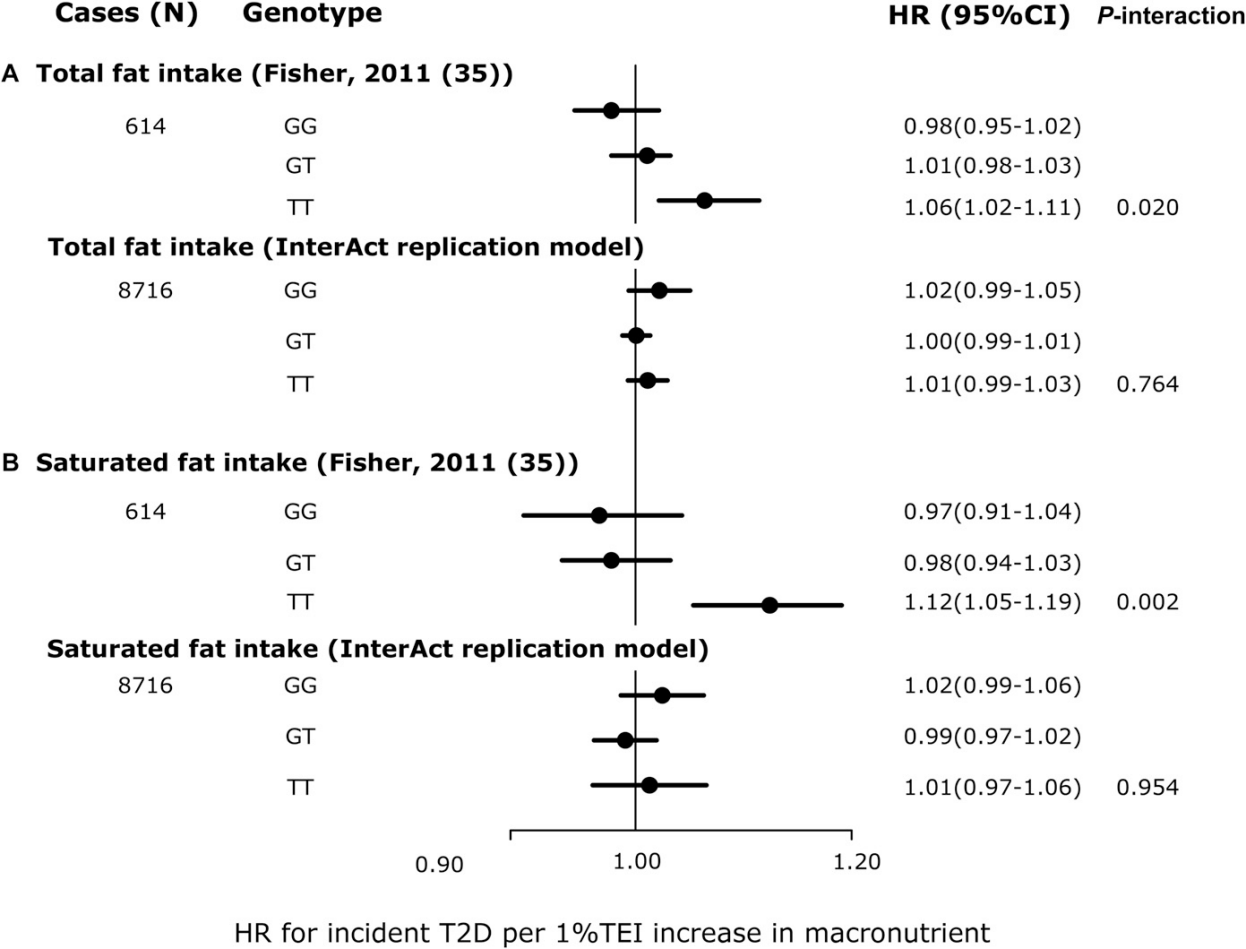
HRs of incident T2D per 1% TEI increase in macronutrient intake, stratified by *CAV2* rs2270188 genotype: comparison between Fisher et al. (35) and EPIC-InterAct. HRs from Fisher et al. (35) (top) and pooled HRs from EPIC-InterAct (bottom) for both total fat intake (A) and saturated fat intake (B). Fisher et al. (35) adjusted for sex, age, TEI, and BMI (*P*-interaction values were obtained using results from the confirmatory case-cohort study under the additive genetic model). The EPIC-Interact replication model was adjusted for age (equal to the underlying time scale), sex, center, TEI, and BMI, excluding the EPIC-InterAct Potsdam center. To note, the classical interaction model was adopted, not the genotype-specific model reported in Fisher et al. (35), because of the stated equivalence of the 2. *P*-interaction values were estimated by treating macronutrients and rs2270188 as continuous variables. In the EPIC-InterAct study, heterogeneity between countries was moderate (*I*^2^ = 41% and 34% in panels A and B, respectively). The total sample size for the EPIC-InterAct analysis was 19,477. Multiplicative interaction analysis was performed using Prentice-weighted Cox regression. *CAV2*, caveolin 2; EPIC, European Prospective Investigation into Cancer; TEI, total energy intake; T2D, type 2 diabetes.

**FIGURE 5:**
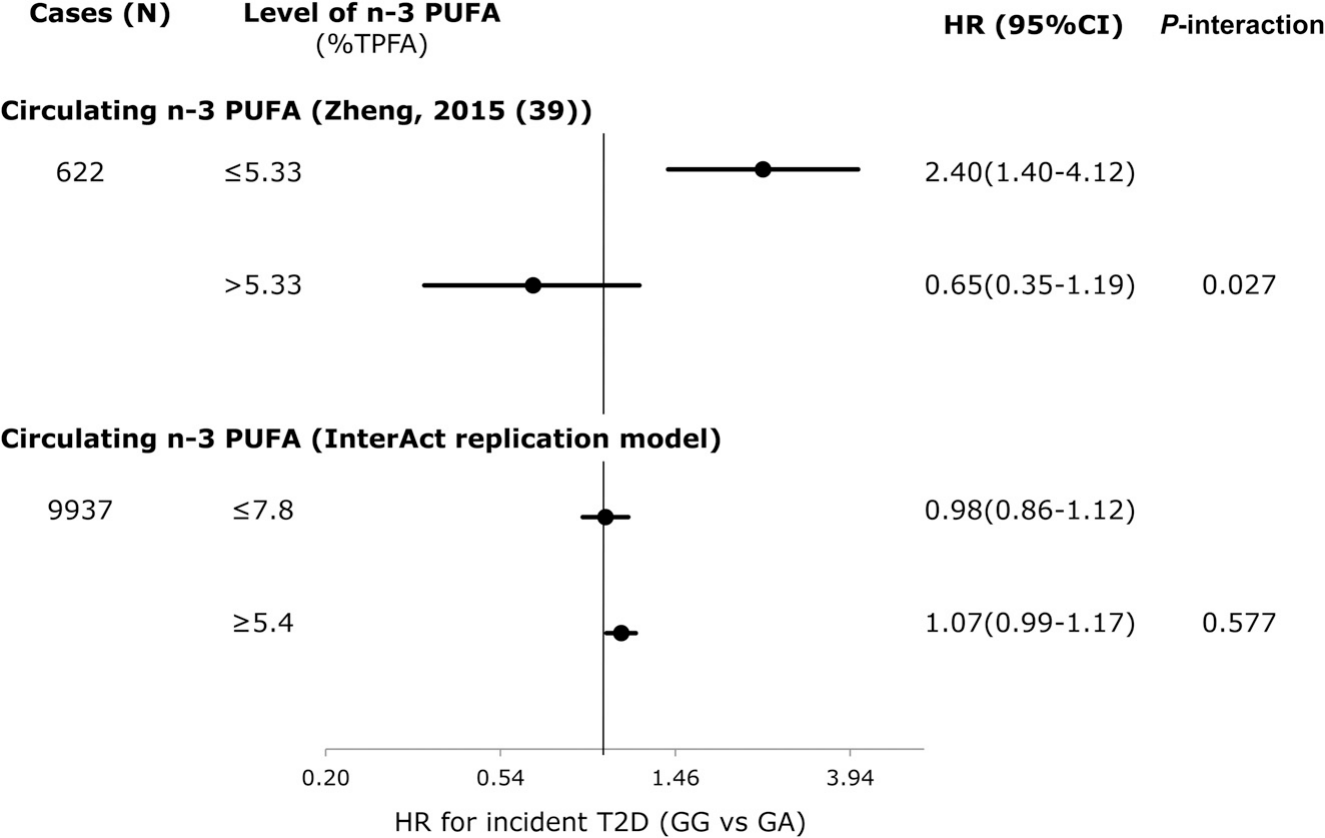
Interaction between genotypes for rs3786897 (*PEPD*: GA vs. GG) and the percentage of TPFAs that are circulating n–3 PUFAs: comparison between Zheng et al. (39) and EPIC-InterAct. ORs from Zheng et al. (39) (top) and pooled HRs from EPIC-InterAct (bottom) for T2D. Zheng et al. (39) adjusted for age and sex. The EPIC-InterAct replication model adjusted for age (equal to the underlying time scale), sex, and center. *P*-interaction values were estimated by treating circulating n–3 PUFAs as dichotomous and *PEPD* rs3786897 as continuous variables. In EPIC-InterAct, heterogeneity between countries was not significant (*I*^2^ =15%). The total sample size for the EPIC-InterAct analysis was 22,273. Multiplicative interaction analysis was performed with Prentice-weighted Cox regression. EPIC, European Prospective Investigation into Cancer; *PEPD*, peptidase D; TEI, total energy intake; TPFA, total phospholipid fatty acid; T2D, type 2 diabetes.

We found no significant interactions for any of the replication analyses in EPIC-InterAct that were comparable to the model specifications in the published literature. **Figure 2**A shows that compared with the original report (*P*-interaction = 0.049) (1649 cases of T2D/24,799 total) (10), we failed to replicate the significant interaction between *TCF7L2* rs7903146 and dietary fiber intakes for incident T2D in EPIC-InterAct (*P*-interaction = 0.97) (8012 cases of incident T2D/18,292 total). The covariates included in each model are detailed in the figure legend. We also did not observe any interaction in EPIC-InterAct by subtypes of dietary fiber (cereal, vegetable, or fruit fiber) (*P*-interaction ≥ 0.27) (**Supplemental Figure 1**). Figure 2B shows no replication of the interaction between *TCF7L2* and GL for the risk of developing T2D (*P*-interaction = 0.58) as previously detected by Cornelis et al. (13). Similarly, we did not detect a significant interaction reported between rs10423928 (in *GIPR*) and carbohydrate or fat intake for incident T2D (*P*-interaction = 0.79 and 0.25, respectively) (41) (**Figure 3**). At *CAV2*, where an interaction was reported between both total fat and SFA intake with rs2270188 (35), we found no evidence to support this in EPIC-InterAct (*P*-interaction = 0.76 and 0.95, respectively) (**Figure 4**). In additional analysis, however, we detected a significant interaction when Potsdam, the center originally analyzed in the previous publication (35), was analyzed independently (*P*-interaction = 1.01 × 10^−6^ and 0.001 for total FAs and SFAs, respectively). The interaction between rs3786897 (within *PEPD*) and circulating n–3 PUFAs reported by Zheng et al. (39) was also not observed in EPIC-InterAct (*P*-interaction = 0.58) (**Figure 5**).

There was also no evidence of any significant interaction in our more detailed analysis that accounted for additional potential confounders and isocaloric macronutrient substitution (**Supplemental Figures** 1**–4**).

## DISCUSSION

We identified 13 articles reporting gene-macronutrient interactions on T2D from our systematic review, but we did not find any consistently replicated evidence for gene-macronutrient interaction in the etiology of T2D.

### Challenges in identifying and replicating gene-macronutrient interactions

Differences observed between findings from the published studies and EPIC-InterAct re-emphasize the challenges in studying gene-diet interactions. Selective reporting through limited consideration for multiple testing in studies examining multiple SNPs and/or macronutrients, without a justified predefined hypothesis and lack of replication, is one of several possible methodologic explanations for this inconsistency. As discussed in previous reviews, other factors that may explain why we find different results from those of the published studies may include heterogeneity in dietary measurement, study population, study design, or analysis and reporting (14, 47–49).

Given the large number of variants tested on a genome-wide scale, stringent correction for multiple testing in hypothesis-free genetic epidemiologic analyses has attempted to minimize the false-positive rate (50). However, approaches for interaction studies have been less consistent. We found in our review that studies often used a nominal *P* < 0.05 as the threshold for rejecting the null, even when performing many tests (10, 11, 13, 35, 41). For example, 1 study performed an exploratory analysis of 256 gene-macronutrient interactions and used *P* < 0.05 for rejecting the null (35). Two of the 6 studies that reported significant interactions would have passed multiple testing corrections after Bonferroni correction (11, 41), whereas 1 study adopted Bonferroni-corrected *P* values (39). Therefore, we consider false-positive reports as a potential explanation for the discordant findings between EPIC-InterAct and published reports. Although debate continues about whether an optimal *P* value threshold should exist for interaction studies (51), researchers should account for potential inflation of a false-positive rate when conducting multiple-interaction analyses in the future (e.g., by using methods such as the “effective number of independent tests”) (52), preferably with independent replication in additional studies. As evidenced by genome-wide association studies, the design of genetic studies allows for relatively straightforward in silico replication, yet few gene-macronutrient interaction studies have been followed with independent replication (10, 11, 13, 39, 41). Arguably, variations in dietary assessment methods introduce more difficulty in identifying suitable replication sources. For instance, although 4 independent studies included in our review examined the interaction between *TCF7L2* and dietary fiber or related fiber subtypes (10, 11, 13, 36), it is arguable how comparable their methods are. For example, in relation to dietary assessment and degree of measurement error: 2 studies used an FFQ (11, 13), 1 used a 24-h recall (36), and 1 used a combined FFQ, diet history, and 7-d diary (10). There were also differences in study design: 2 studies were prospective (10, 11) and 2 were cross-sectional (13, 36), which may be subject to differing levels of bias and ability to determine the direction of effect. Finally, analytic methods varied by whether variables were treated as continuous or categorical and what covariates were controlled for. However, internally conducted replication would reduce variation in analysis. Researchers may consider both observational or intervention settings, in which genotype-driven recruitment methods may aid in maximizing statistical power (53).

We tried to mirror the population and analyses conducted in EPIC-InterAct with those of the published studies reporting an interaction, and we showed comparable characteristics except with one study in which ethnicity was different (an Asian population was examined) (39). However, we cannot exclude possible heterogeneity between studies. This may include differences in study design (only one published study used a case-cohort study design similar to EPIC-InterAct) and unmeasurable inconsistencies in dietary exposures (e.g., food composition, preparation methods, measurement tool used, coding of exposures) between countries within EPIC-InterAct and between EPIC-InterAct and the published studies. Indeed, this was evident for the interaction between *CAV2* with total fat and SFAs, which showed center specificity. Within the German centers, an interaction was detected for the Potsdam EPIC-InterAct center but not Heidelberg, resulting in an overall lack of interaction for Germany. However, the percentage of total variation attributable to heterogeneity across the countries within EPIC-InterAct was low to moderate for interactions under the replication model (*I*^2^: 14–30%). The consistently null findings across different countries of EPIC-InterAct strengthen the inference from this overall null finding. Another possible contributor to the disparity between results (e.g., relating to *TCF7L2* and dietary fiber) may be overestimation by certain estimation parameters (e.g., ORs), which could lead to an inflated difference between fiber categories (54).

The methodologic issues described above highlight difficulties in discerning whether type I error or true heterogeneity underlies the inconsistencies we observed and are similar to those faced in the broader gene-environmental literature (51, 55). For gene-environment interactions, recommendations have been made for improving standards in design, analysis, and reporting, which are also relevant for gene-diet studies (14, 24). For example, Cornelis suggested minimizing publication bias by publishing both positive and negative interaction findings and reporting them in supplemental materials if necessary (14).

### Strengths and limitations

A potential limitation of our systematic review is that the heterogeneity between the published studies (i.e., in study design, statistical analysis, and reporting) did not enable a quantitative synthesis (e.g., meta-analysis) or formal statistical evaluation of publication bias, as previously demonstrated by Palla et al. (20). We did, however, use a comprehensive search strategy and attempted to minimize publication bias by contacting authors of studies possibly examining interactions (*n* = 4).

As the largest study of incident T2D cases (>5 times that of previous studies) with both genetic data as well as measures of self-reported macronutrient intake and objective circulating FAs to date, EPIC-InterAct is well positioned to examine these reported interactions (power calculations available in **Supplemental Table 6**). The prospective design minimizes the potential bias owing to reverse causality for dietary exposures. In addition, to our knowledge, this is the first study of gene-macronutrient interactions that has investigated the effect of isocaloric macronutrient substitution in the observational setting. This is important for public health interpretation of macronutrient density if total daily energy intake is fixed, because the benefit of decreasing one macronutrient may be dependent on which macronutrient replaces it. Several limitations must be considered while interpreting these results. Our analyses only investigated a select number of interactions that have been reported in the literature. Hence, this does not preclude the possibility that there may be interactions between other dietary factors (including foods and dietary patterns) and other genes or combined gene scores. Moreover, our focus was on examining possible type I error. Given that we did not examine interactions that did not reach statistical significance in published studies (possible type II error), we cannot preclude the presence of genuine interactions among those loci we did not test. Alternative study designs may be better suited to investigate the presence of these interactions (56). Variations in dietary assessment between EPIC-InterAct centers may contribute to potential variation in measurement error for macronutrients. The current literature consists of studies primarily from European populations, which limits the generalizability of our findings.

### Implications for public health and research

Our study highlights the importance of independent replication in studying interactions and the need to improve standards in conducting and reporting future interactions. Moreover, our review reveals a gap in noncandidate gene approaches to examining gene-macronutrient interactions. This includes genetic risk scores and genome-environment–wide interaction studies. Given that we found no promising gene-macronutrient interactions and that genetic variants most relevant for interactions may be those with weak or no marginal effects (7, 57), genome environment–wide interaction studies may aid in discovering novel interactions at potentially unexpected genetic loci. Furthermore, we highlight that on the basis of the interactions examined here, there is currently no evidence to support genetic personalization of macronutrient intake recommendations as a strategy to prevent T2D.

Based on the issues highlighted in our review, we recommend that investigators consider the following in future research examining gene-macronutrient interactions. Within-study considerations include *1*) specifying the hypothesis of the study and accounting for multiple testing, as appropriate; *2*) reporting all interaction results and whether they were analyzed as preplanned or post hoc, regardless of whether findings are positive, negative, or null; and *3*) ensuring that notable interaction findings are accompanied by independent replication where possible (if this is not feasible, the reasons for and validity of nonreplicated findings should be discussed). General considerations for studies within the field include *1*) improving consistency and standards in examining and reporting interactions (14, 24, 58), *2*) conducting studies examining non-European populations, and *3*) applying isocaloric macronutrient substitution.

In conclusion, although there is growing interest in personalized diets to more effectively combat T2D, none of the gene-macronutrient interactions currently reported in the literature could be replicated in a large-scale EPIC-InterAct study. Improving standards in examining and reporting interactions, including independent replication, will be vital to making progress in this area.
